# Histopathological findings in laparoscopic sleeve gastrectomy specimens and their relation to preoperative endoscopy: A retrospective cross-sectional study of 954 patients

**DOI:** 10.1097/MD.0000000000051246

**Published:** 2026-10-23

**Authors:** Moaz Abulfaraj, Murad M. Aljiffry, Mohammed F. Alhazmi, Ashraf A. Maghrabi, Rana Y. Bokhary, Abdulrahman Faihan A. Alotaibi, Rania A. Fouad, Mohamed El-Sherbiny, Hasnaa Ali Ebrahim, Tagwa Salah Ahmed Mohammed Ali, Dalia Mahmoud Abdelmonem Elsherbini

**Affiliations:** a Department of Surgery, Faculty of Medicine, King Abdulaziz University, Jeddah, Saudi Arabia; b Department of Pathology, Faculty of Medicine, King Abdulaziz University, Jeddah, Saudi Arabia; c Surgery Department, King Faisal Medical Complex, Taif, Saudi Arabia; d Department of Basic Medical Sciences, College of Medicine, AlMaarefa University, Riyadh, Saudi Arabia; e Department of Medical Biochemistry, College of Medicine, Zagazig University, Zagazig, Egypt; f Research Center, Deanship of Scientific Research and Post-Graduate Studies, AlMaarefa University, Riyadh, Saudi Arabia; g Department of Basic Medical Sciences, College of Medicine, Princess Nourah bint Abdulrahman University, Riyadh, Saudi Arabia; h Department of Clinical Laboratory Sciences, College of Applied Medical Sciences, Jouf University, Sakaka, Saudi Arabia; i Department of Parasitology and Medical Entomology, Faculty of Medical laboratory Science, Sudan University of Science and Technology, Khartoum, Sudan; j Department of Anatomy, Faculty of Medicine, Mansoura University, Mansoura, Egypt.

**Keywords:** esophagogastroduodenoscopy, gastritis, helicobacter pylori infection, histopathological examination, laparoscopic sleeve gastrectomy

## Abstract

Laparoscopic sleeve gastrectomy (LSG) is the most frequently performed bariatric surgery. Although it is generally considered safe, a thorough preoperative assessment is essential to minimize the risk of complications. The need for histopathological examination of resected gastric specimens in bariatric surgery remains a subject of debate, particularly regarding the role of preoperative esophagogastroduodenoscopy (EGD). This study aimed to determine the prevalence of histopathological findings in resected specimens following sleeve gastrectomy and to assess their agreement with preoperative EGD findings at a tertiary center. This retrospective cross-sectional study examined patients who underwent LSG between 2009 to 2021. The data included demographics, comorbidities, preoperative EGD findings, and routine postoperative histopathology of the resected specimens. Diagnostic accuracy of preoperative EGD was assessed against resected-specimen histopathology. Among the 954 patients who underwent LSG, 67.1% were female, and 84.7% were Saudi nationals. Abnormal histopathological findings were observed in 45.5% (434/954) of the resected specimens, with gastritis being the predominant finding, present in 98.2% (426/434) of these. Preoperative EGD was performed in a subgroup of 357 patients (37.4%); in these, gastritis was the most prevalent EGD finding (50.1%). Helicobacter pylori was identified in 9.5% of patients who underwent preoperative EGD and in 8.0% of all resected specimens. Less common histopathological findings were metaplasia (0.6%), gastrointestinal stromal tumors (0.3%), gastric polyps (0.2%) and grade 1 gastric carcinoid (0.1%). Among the 357 patients who underwent preoperative EGD, sensitivity was 99.5%, specificity 98.3%, positive predictive value 98.4%, negative predictive value 99.4% and accuracy 98.9% against resected-specimen histopathology. Gastritis is the most prevalent histopathological finding in resected specimens following sleeve gastrectomy and on preoperative EGD, although significant pathologies are infrequent. In the selected subgroup of patients who underwent preoperative EGD, a normal EGD showed high agreement with normal histopathology of the resected specimen; these findings do not support omitting histopathological examination of resected specimens, and prospective multicenter studies are needed. Helicobacter pylori was frequently identified and was strongly associated with gastritis in unadjusted analyses; premalignant findings were too few to assess.

## 1. Introduction

Bariatric surgery is recognized as the most effective strategy for addressing morbid obesity and related comorbidities, outperforming lifestyle modifications, pharmacological treatments, and endoscopic techniques. Bariatric surgery can be classified into 2 primary methods: restrictive and malabsorptive.^[1]^

Laparoscopic Sleeve Gastrectomy (LSG) is currently the most used method for bariatric surgery on a global scale.^[2–5]^ The procedure exhibits a restrictive nature and is favored over malabsorptive procedures, such as gastric bypass or duodenal switch. These 2 alternatives pose technical difficulties and are associated with a greater likelihood of vitamin and protein deficiencies. The LSG method is characterized by its simplicity and ability to produce outstanding outcomes. It involves surgical removal of approximately 80% of the stomach while preserving the pylorus, resulting in the formation of a new, sleeve-shaped stomach. Although LSG is generally regarded as a safe and replicable surgical procedure, it is not completely free of complications. However, these can be severely mitigated through appropriate preoperative assessments and preparation.^[2–5]^

In the existing literature, the prevalence of abnormal findings during preoperative esophagogastroduodenoscopy (EGD) prior to bariatric surgery has been documented to vary between 4.6% and 89.7%.^[6]^ For instance, a study revealed that preoperative EGD findings of benign pathologies, such as treatment for Helicobacter pylori (H. pylori) infection and gastritis, led to a 16.8% alteration in the surgical approach.^[6]^ A separate study revealed that only 8% of endoscopic findings led to modifications in the surgical treatment of bariatric patients.^[7]^ Additionally, owing to the relatively low occurrence of gastric malignancy (0.4%) and premalignant alterations (2.2%),^[8]^ several hospitals do not routinely submit resected gastric specimens for pathological assessment after SG. It is not commonly performed as a standard practice, primarily because of concerns regarding costs and the need for insurance approval. Assessment of gastric condition is frequently overlooked, particularly in patients who have not undergone preoperative EGD.

The role of preoperative EGD in patients undergoing bariatric surgery remains debated. Its primary objective is to detect pathology that may influence surgical decision-making, including Barrett’s esophagus and H. pylori infection. The 2020 EAES guideline gives a conditional recommendation for routine esophagogastroscopy before bariatric surgery, noting that selective endoscopy in symptomatic patients may be a reasonable alternative given the low certainty of the evidence.^[9]^ Regarding *H. pylori*, the same guideline found insufficient evidence to favor either routine eradication or standard care, and therefore issued a conditional recommendation for either approach.^[9]^ The 2021 ASMBS position statement similarly leaves routine preoperative EGD to the surgeon’s discretion, while highlighting its role in detecting esophageal mucosal injury particularly Barrett’s esophagus given the recognized risk of GERD progression after sleeve gastrectomy.^[10]^ The 2025 MAPS III guideline further supports *H. pylori* eradication specifically in patients with precancerous gastric conditions or after endoscopic/surgical therapy.^[11]^ Together, these guidelines support an individualized, risk-stratified approach to preoperative EGD rather than a uniform mandate.

This study aimed to characterize the spectrum and prevalence of histopathological findings in resected gastric specimens obtained from patients who underwent sleeve gastrectomy. Furthermore, we aimed to assess the agreement between preoperative EGD findings and the histopathological results of the resected specimens following sleeve gastrectomy.

## 2. Methods and materials

### 2.1. Study design and setting

This was a retrospective, cross-sectional study. Data were extracted from the electronic medical records of patients who underwent sleeve gastrectomy between 2009 and 2021. The medical records of the 982 patients who underwent LSG were assessed for completeness. Twenty-eight records were excluded due to incomplete data. Consequently, 954 patients underwent LSG, and a histopathological examination of the resected specimens was conducted. Ethical approval was obtained from the Unit of Biomedical Ethics, Research Committee at King Abdulaziz University, Faculty of Medicine (Reference No. 479-21). The study adhered to the Declaration of Helsinki (2013 revision)^[12]^ and is reported according to the Strengthening the Reporting of Observational Studies in Epidemiology (STROBE) guidelines for observational studies.^[13]^ The requirement for informed consent was waived by the committee because of the retrospective design and the use of anonymized data.

### 2.2. Participants:

Patients who underwent LSG were categorized as overweight (25-29.9 kg/m^2^), obesity class 1 (30–34.9 kg/m^2^), obesity class II (35–39.9 kg/m^2^), and obesity class III (>40 kg/m^2^). Exclusion criteria encompassed patients with incomplete data in the retrieved medical records.

### 2.3. Data sources/measurement

Data collection included various variables such as age, sex, nationality, body mass index (BMI), comorbidities, length of hospitalization, preoperative EGD findings, preoperative and postoperative medical records, and histopathological analysis of resected specimens. Data were extracted using an accurately generated data sheet that was aligned with the specific objectives of the study. The extracted data were stored in an Excel spreadsheet on a protected computer system to maintain patient confidentiality. Operative risk was classified using the American Society of Anesthesiologists (ASA) physical status classification system.^[14]^ A specimen could carry more than 1 diagnosis, and each diagnosis was counted in its own category.

### 2.4. Study sample

No formal sample-size calculation was performed. The study is a census of all patients who underwent LSG at the center between 2009 and 2021 and had complete records (982 assessed; 28 excluded for incomplete data; 954 analyzed). The precision of the main prevalence estimates is reported as 95% confidence interval (CI); for example, abnormal histopathology was present in 434/954 (45.5%; 95% CI 42.3–48.7), and EGD was performed in 357/954 (37.4%; 95% CI 34.3–40.6).

### 2.5. Statistical methods

Data were analyzed using IBM SPSS Statistics version 25 (IBM Corp., Armonk). Firth-penalized logistic regression and exact CIs were calculated in R version 4.1.3 (R Foundation for Computing, Vienna, Austria) with the logistf package, and forest plots were drawn with GraphPad Prism [version 8] (GraphPad Software, San Diego). Continuous variables are summarized as mean and standard deviation (SD), and categorical variables as frequency and percentage. Prevalences are reported with exact (Clopper–Pearson) 95% CIs. All tests were two-sided. Diagnostic accuracy of preoperative EGD was assessed in patients who underwent EGD, with abnormal resected-specimen histopathology as the reference standard and target condition. Sensitivity, specificity, positive and negative predictive values, and accuracy were calculated from the 2 × 2 table with exact (Clopper–Pearson) 95% CI; agreement was assessed with Cohen’s kappa, and the association between EGD and histopathology was tested with Fisher’s exact test. Unadjusted associations between candidate factors (age >55 years, female sex, BMI > 39.9 kg/m^2^, hypertension, diabetes mellitus, hypothyroidism, dyslipidemia, and *H. pylori*) and histopathological outcomes with adequate numbers of events (gastritis on EGD and mild chronic inactive, mild chronic active, moderate chronic active, and follicular gastritis) were explored using odds ratio (OR) with 95% CIs and Fisher’s exact test. Where 1 cell contained no events, a Firth-penalized OR with a profile-likelihood 95% CI was calculated; when fewer than 1 event was expected in the exposed group, no OR is reported. Each outcome was treated as a separate family of 8 hypotheses, and multiplicity within a family was controlled by the Bonferroni correction (adjusted *α* = 0.05/8 = 0.00625), which controls the family-wise error rate. Results with *P* < .05 but ≥ 0.00625 are reported as nominal associations, and results with *P* < .00625 as significant after adjustment. The 95% CIs are nominal (not adjusted for multiplicity), and these analyses are exploratory. Uncommon findings are reported descriptively with exact 95% CIs. No hypothesis tests were applied to baseline or descriptive tables.

## 3. Results

### 3.1. Baseline characteristics of the study group

A total of 954 patients underwent LSG and subsequent histopathological evaluation of resected specimens (Fig. 1). Within this group, 67.1% of the patients were female and 32.9% were male. The study revealed that the majority of the participants were Saudi citizens, comprising 84.7% of the total sample. The mean ± SD age of the participants was 39.1 ± 10.62 years. The average BMI of the participants was 44.1, with a SD of 11.4. Most patients were classified as obese class III (62.9%), followed by class II (24.2%). The most prevalent comorbidities were hypertension (14.3%) and diabetes mellitus (10.9%). Most patients had operative ASA scores of I and II at 25.9% and 63.6%, respectively, as detailed in Table 1.

**Table 1:** Baseline demographics of the study group (n = 954).

Variables	Mean ± SD or n (% of all 954 patients)
Age (yr)	39.18 ± 10.62
< 35	354 (37.1%)
35–55	511 (53.6%)
>55	89 (9.3%)
Sex	
Male	314 (32.9%)
Female	640 (67.1%)
Nationality	
Saudi	808 (84.7%)
Non-Saudi	146 (15.3%)
BMI (kg/m^2^)	44.1 ± 11.4
Overweight (25–29.9)	44(4.6%)
Obesity Class I (30–34.9 kg/m^2^)	79 (8.3%)
Obesity Class II (35–39.9 kg/m^2^)	231(24.2%)
obesity class III (> 40 kg/m^2^)	600(62.9%)
Associated comorbidities (number with the condition)	
Hypertension	136(14.3%)
Diabetes mellitus	104(10.9%)
Hypothyroidism	50 (5.2%)
Bronchial asthma	26 (2.7%)
Dyslipidemia	25 (2.6%)
Obstructive sleep apnea	21 (2.2%)
Joint disorders	12(1.3%)
Ischemic Heart Disease	7 (0.7%)
Mild gastroesophageal reflux disease	7 (0.7%)
Polycystic Ovarian Disease (PCOD)	4(0.4%)
Breast cancer	2 (0.2%)
Pituitary adenoma	2 (0.2%)
Cardiac dysrhythmia	2 (0.2%)
Hiatal hernia	2 (0.2%)
ASA physical status classification system (operative risk (ASA score)	
ASA I	247 (25.9%)
ASA II	607 (63.6%)
ASA III	100 (10.5%)

Continuous variables are mean ± SD; categorical variables are n (%).No hypothesis tests were performed.

ASA = American Society of Anesthesiologists, BMI = body mass index.

**Figure 1.**
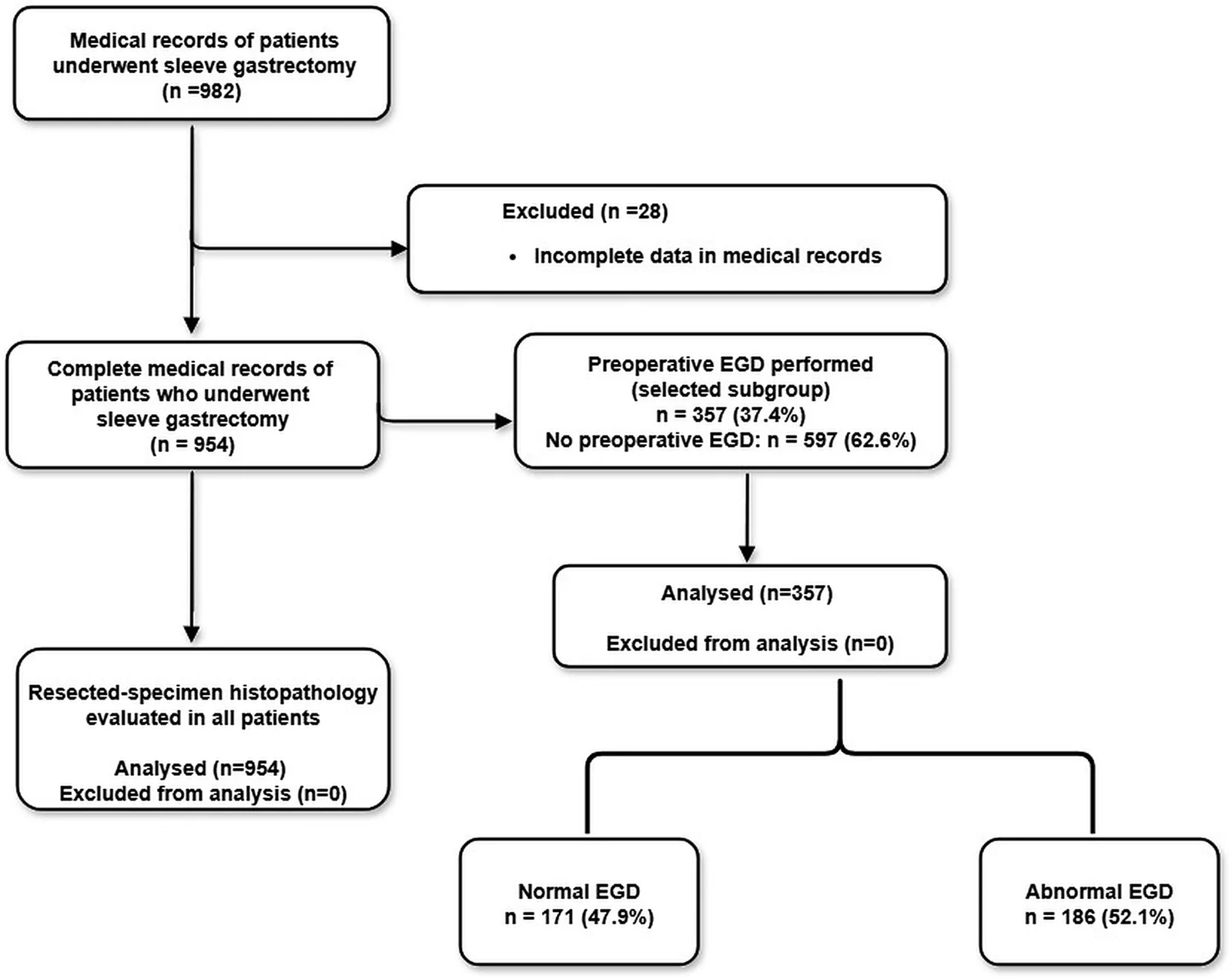
Flow of patients. Of 982 records assessed, 28 were excluded for incomplete data, and 954 were analyzed. Preoperative EGD was performed in a selected subgroup (n = 357; 171 normal, 186 abnormal) and not in the remaining 597 patients. EGD = Esophagogastroduodenoscopy.

### 3.2. Preoperative EGD

In the current study, among the patients who underwent LSG and had resected specimen histopathology, 37.4% (357/954) underwent preoperative EGD, while the remaining 62.6% (597/954) did not undergo this procedure (Fig. 1). Among patients who underwent preoperative EGD, 47.9% (171/357) exhibited normal results, whereas 52.1% (186/357) presented with abnormal findings. Using abnormal resected-specimen histopathology as the reference standard, preoperative EGD (n = 357) had a sensitivity of 99.5% (95% CI 97.0–100.0), a specificity of 98.3% (95.0–99.6), a PPV of 98.4% (95.4–99.7), an NPV of 99.4% (96.8–100.0), and an accuracy of 98.9% (97.2–99.7) (Table 2). Of 171 normal EGDs, one had abnormal histopathology (mild inactive chronic gastritis); none showed metaplasia, neoplasia, or gastrointestinal stromal tumor (GIST). Among the 357 patients who underwent preoperative EGD, gastritis was the most common abnormality (179, 50.1%). Other observed conditions included H. pylori infection (34, 9.5%), gastric polyps (2, 0.6%), metaplasia (2, 0.6%), and grade 1 gastric carcinoid (1, 0.3%) (Table 3). Of the 34 patients with *H. pylori* at preoperative EGD, 33 (97.1%) were also positive on resected-specimen histopathology, and one was negative. *H. pylori* was diagnosed at preoperative EGD in 34 patients and on resected-specimen histopathology in 35; the 2 measures agreed in 33 patients, with 1 patient positive by EGD only and 1 positive by histology only. Among the 186 patients with abnormal EGD findings, the preoperative hospital stay was 1 day in most (150/186; range 1–8 days). The longest mean stay (5 days) was in the single patient with focal mucosal ischemic changes (Table 3).

**Table 2:** Preoperative EGD *versus* resected-specimen histopathology (n = 357).

*A. Cross-tabulation of EGD findings and histopathology*			
	Abnormal histopathology	Normal histopathology	Total
Abnormal EGD	183	3	186
Normal EGD	1	170	171
Total	184	173	357

B. Diagnostic accuracy of preoperative EGD			
Metric (target: abnormal histopathology)	Value		95% CI (Clopper–Pearson)
Sensitivity	99.5% (183/184)		97.0–100.0
Specificity	98.3% (170/173)		95.0–99.6
PPV	98.4% (183/186)		95.4–99.7
NPV	99.4% (170/171)		96.8–100.0
Accuracy	98.9% (353/357)		97.2–99.7

CI = confidence interval, EGD = esophagogastroduodenoscopy, NPV = negative predictive value, PPV = positive predictive value.

Reference standard: histopathology of the resected specimen; target condition: abnormal histopathology. Confidence intervals are exact (Clopper–Pearson).

The positive likelihood ratio is 57.4 and the negative likelihood ratio is 0.006. Cohen’s kappa is 0.97, and Fisher’s exact test gives *P* < .001.

**Table 3:** Abnormal preoperative EGD findings and their prevalence among patients who underwent preoperative EGD.

Preoperative EGD findings	n (% of the 357 patients who underwent preoperative EGD)	Pre-operative hospital stays length (d) median (IQR) [range]
Any abnormal finding	186 (52.1%)	1 (1–1) (1–8)
Gastritis	179 (50.1%)	1(1–1)(1–8)
*Helicobacter pylori*	34 (9.5%)	1(1–1)(1–4)
Gastric polyp	2 (0.6%)	1(1–1)
Metaplasia	2 (0.6%)	1(1–1)
Gastric carcinoid grade 1	1 (0.3%)	1(single patient)
Focal erosion	1 (0.3%)	1(single patient)
Focal mucosal ischemic changes	1 (0.3%)	5(single patient)

n (%): percentage of the 357 patients who underwent preoperative EGD. Categories are not mutually exclusive (all H. pylori cases are also counted as gastritis), so rows do not sum to the total. Length of stay is median (interquartile range) [range]; for groups of 1 or 2 patients only the value or range is shown. No hypothesis tests were performed.

EGD = esophagogastroduodenoscopy, IQR = interquartile range.

Figure 2 shows unadjusted OR for candidate factors associated with gastritis among patients who underwent EGD. All 35 patients with *H. pylori* in the resected specimen had gastritis on EGD, so a Firth-penalized OR is reported (OR 87.7, 95% CI 12.3–11,132.0; *P* <.001), which remained significant after Bonferroni adjustment (*α* = 0.00625), although the interval is extremely wide. No other factor was significant: BMI >39.9 kg/m2 (OR 1.27, 95% CI 0.81–1.93; *P* = .32), age >55 years (OR 1.26, 95% CI 0.65–2.53; *P* = .61), and dyslipidemia (OR 0.54, 95% CI 0.18–1.64; *P* = .29) (Supplementary Table 1, Supplemental Digital Content 1**).**

**Figure 2.**
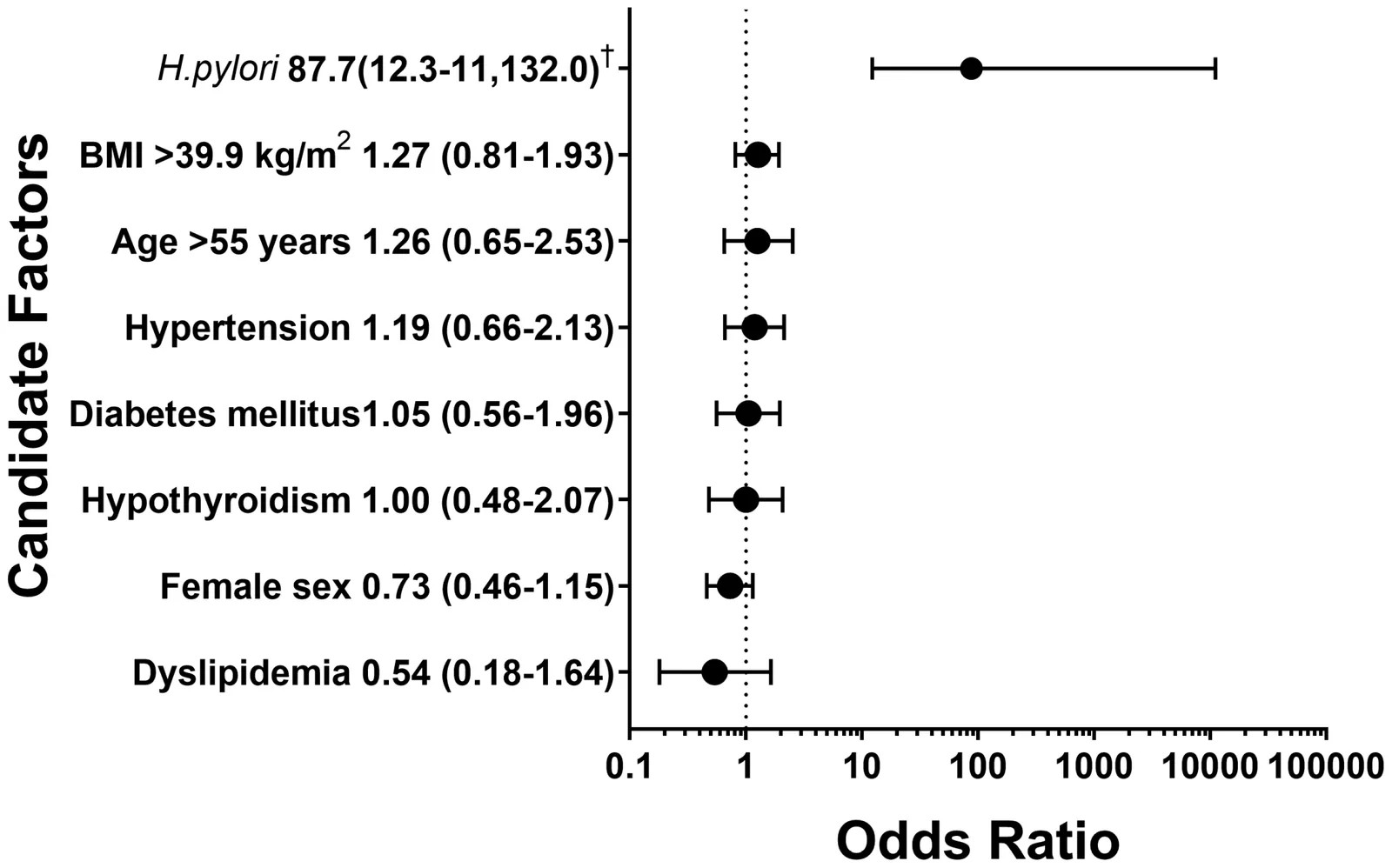
Unadjusted odds ratios (OR) with nominal 95% CIs for candidate factors associated with gastritis on preoperative EGD (179/357). *H. pylori* = present in the resected specimen. Fisher’s exact test; Bonferroni-adjusted threshold for 8 comparisons, *α* = 0.00625. †Firth-penalized estimate (all 35 patients with *H. pylori* had gastritis), with a profile-likelihood 95% CI. BMI = body mass index, EGD = Esophagogastroduodenoscopy, H. pylori = Helicobacter pylori, OR = odds ratio.

Operative risk, as indicated by an ASA score of II, was prevalent among patients with abnormal preoperative EGD findings (Table 4).

**Table 4:** Operative risk (ASA score) by preoperative EGD finding among patients who underwent preoperative EGD.

Preoperative EGD findings	ASA I	ASA II	ASA III
	n (% among patients who underwent preoperative EGD)		
**Any abnormal finding**	**64 (17.9%**)	**104 (29.1%**)	**18 (5.0%**)
Gastritis	60 (16.8%)	103 (28.9%)	16 (4.5%)
*Helicobacter pylori*	12 (3.4%)	22 (6.2%)	0 (0.0%)
Gastric polyp	0 (0.0%)	0 (0.0%)	2 (0.6%)
Metaplasia	1 (0.3%)	1 (0.3%)	0 (0.00%)
Gastric carcinoid grade 1	1 (0.3%)	0 (0.00%)	0 (0.00%)
Focal erosion	1 (0.3%)	0 (0.00%)	0 (0.00%)
Focal mucosal ischemic changes	1 (0.3%)	0 (0.00%)	0 (0.00%)

n (%): percentage of the 357 patients who underwent preoperative EGD (not of each ASA class). Categories are not mutually exclusive (all *H. pylori* cases are also counted as gastritis). No hypothesis tests were performed.

ASA = American Society of Anesthesiologists, EGD = esophagogastroduodenoscopy.

### 3.3. Histopathological examination of the resected specimens

Within the entire patient group, 54.5% (520/954) demonstrated normal histopathological findings in the resected specimens, whereas 45.5% (434/954) exhibited abnormal findings. The most prevalent histopathological abnormality was gastritis, which accounted for 98.2% (426/434) of the abnormal specimens (44.7% of all patients). Gastritis was categorized as mild or moderate, based on the extent of chronic inflammatory cell infiltration in the lamina propria. Percentages below are of all 954 patients (Table 5). Mild inactive chronic gastritis was the most common (22.6%), followed by mild active chronic gastritis (10.8%), follicular gastritis (8.7%; lymphoid follicles with germinal centers in the lamina propria), moderate chronic active gastritis (2.1%), and chronic atrophic gastritis (0.2%).

**Table 5:** Abnormal resected-specimen histopathological findings and their prevalence.

Resected-specimen histopathological findings	n (% of all 954 patients)	Exact 95% CI	Postoperative hospital stay, d: median (IQR) [range]
Any abnormal finding	434 (45.5%)	42.3–48.7	3 (2–3) [1–45]
Gastritis (any subtype)	426 (44.7%)	41.5–47.9	3 (2–3) [1–45]
Mild chronic inactive gastritis	216 (22.6%)	20.0–25.4	3 (2–3) [1–31]
Mild chronic active gastritis	103 (10.8%)	8.9–12.9	3 (2–3) [1–45]
Follicular gastritis	83 (8.7%)	7.0–10.7	3 (2–3) [1–32]
Moderate chronic active gastritis	20 (2.1%)	1.3–3.2	3 (2–3) [1–7]
Chronic atrophic gastritis	2 (0.2%)	0.0–0.8	2 [2–2]
*Helicobacter pylori*	76 (8.0%)	6.3–9.9	3 (2–4) [1–19]
Metaplasia	6 (0.6%)	0.2–1.4	2 [1–4]
GIST	3 (0.3%)	0.1–0.9	3 [2–3]
Gastric polyp	2 (0.2%)	0.0–0.8	2.5 [2–3]
Hyperplasia	2 (0.2%)	0.0–0.8	2 [2–2]
Focal erosion	1 (0.1%)	0.0–0.6	3 (single patient)
GC grade 1	1 (0.1%)	0.0–0.6	5 (single patient)
Focal mucosal ischemic changes	1 (0.1%)	0.0–0.6	8 (single patient)

n (%): percentage of all 954 patients who underwent laparoscopic sleeve gastrectomy, with exact (Clopper–Pearson) 95% confidence intervals. Categories are not mutually exclusive: a specimen could show more than one finding (for example gastritis with *H. pylori*, metaplasia or hyperplasia), so rows do not sum to the total. Two patients with follicular gastritis plus metaplasia or focal erosion (one each) are counted under the additional finding only. Postoperative hospital stay is median (interquartile range) [range]; for groups of fewer than 10 patients, median [range] only; for a single patient, the value. No hypothesis tests were performed. GC grade 1: gastric carcinoid grade 1.

GIST = gastrointestinal stromal tumor, IQR = interquartile range.

*H. pylori* was identified in 76 patients (8.0%). Several rare observations were noted, including metaplasia (n = 6; 0.6%), GIST (n = 3; 0.3%), gastric polyps (n = 2; 0.2%), hyperplasia (n = 2; 0.2%), focal erosion (n = 1; 0.1%), gastric carcinoid (GC) grade 1 (n = 1; 0.1%), and focal mucosal ischemic changes (n = 1; 0.1%). Findings were not mutually exclusive: 82 patients (8.6% of all patients; 18.9% of the abnormal specimens) had more than one finding, for example, gastritis with *H. pylori* (Supplementary Table 2, Supplemental Digital Content 2**).**

Three patients were found to have GIST in their resected specimens following sleeve gastrectomy. Two of these patients were classified as obesity class II, and one was classified as obesity class III. None of the patients had undergone preoperative EGD. One patient, classified as obese class I with GC grade 1, was diagnosed with preoperative EGD. All 5 patients with polyps (n = 2), metaplasia (n = 2), or grade 1 gastric carcinoid (n = 1) detected on preoperative EGD subsequently underwent LSG, and the same lesion was confirmed in each resected specimen. All tumors were successfully excised, eliminating the need for further therapeutic intervention. Postoperative hospital stay in patients with abnormal specimens was a median of 3 days (IQR 2–3; range 1–45) (Table 5).

### 3.4. Factors associated with gastritis subtypes (unadjusted analyses)

Chronic active gastritis is a prevalent histopathological anomaly that frequently precedes peptic ulcer development. Figure 3 shows unadjusted OR for candidate factors associated with mild chronic inactive gastritis. Hypertension showed a nominal association (OR 1.65, 95% CI 1.11–2.46; unadjusted *P* = .02) that was not significant after Bonferroni adjustment (*α* = 0.00625). No other factor was associated, including diabetes mellitus (*P* = .17), dyslipidemia (*P* = .48), and age >55 years (OR 0.64, 95% CI 0.36–1.11; *P* = .15) (Supplementary Table 3, Supplemental Digital Content 3).

**Figure 3.**
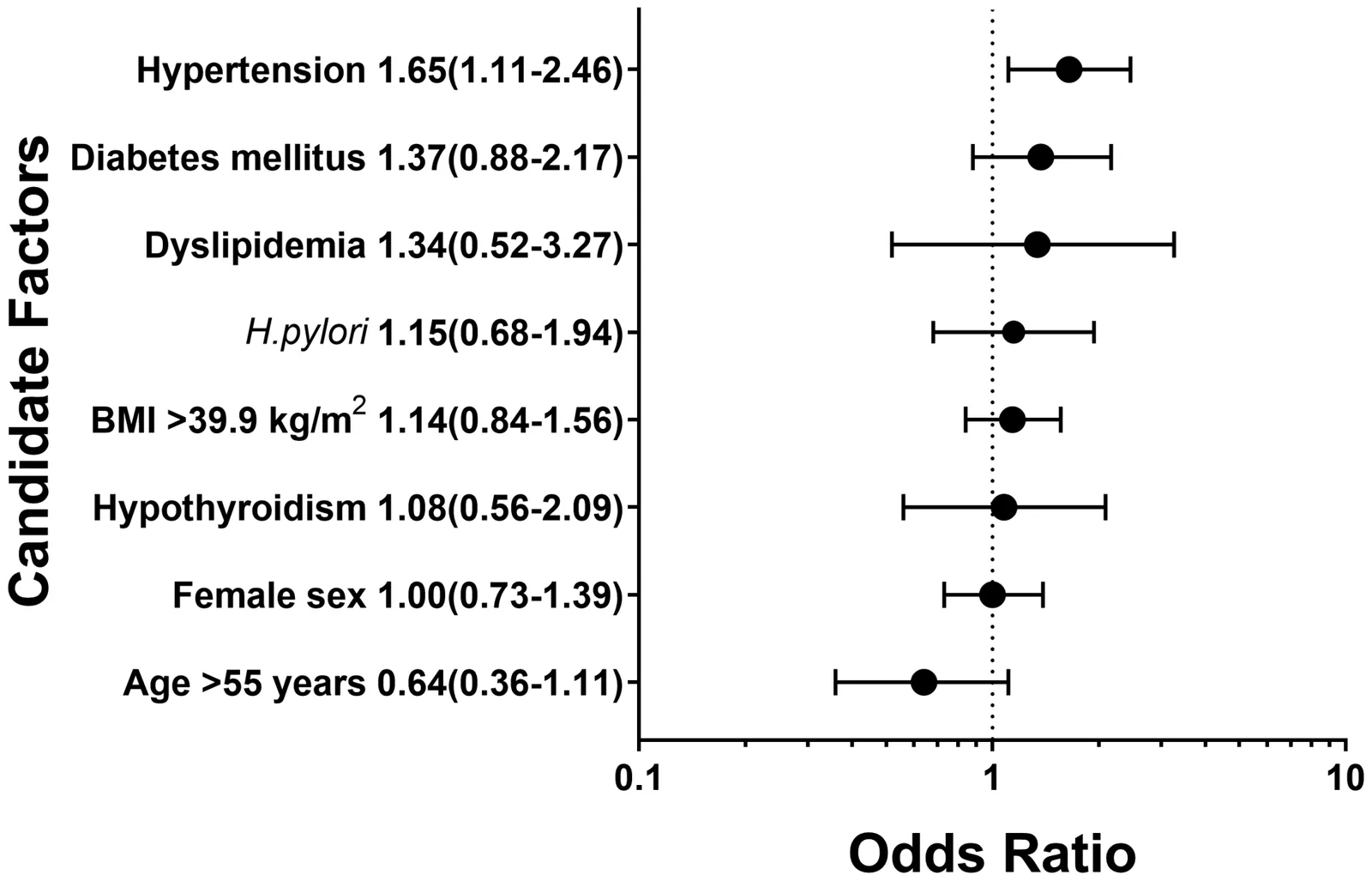
Unadjusted odds ratios (OR) with nominal 95% CIs for candidate factors associated with mild chronic inactive gastritis (216/954). *H. pylori* = present in the resected specimen. Fisher’s exact test; Bonferroni-adjusted threshold for 8 comparisons, α = 0.00625. BMI = body mass index, H. pylori = Helicobacter pylori, OR = odds ratio.

Figure 4 shows unadjusted OR for mild chronic active gastritis. *H. pylori* was strongly associated with this outcome (OR 11.67, 95% CI 6.19–19.69; *P* < .001), which remained significant after Bonferroni adjustment (*α* = 0.00625). Because *H. pylori* and gastritis were identified in the same specimen, this reflects co-occurrence rather than a risk relationship. No other factor was significantly associated (for example, hypothyroidism, *P* = .24). Dyslipidemia was not associated with this outcome (1/25 vs 102/929 without dyslipidemia; OR 0.34, 95% CI 0.05–2.52; *P* = .51) (Supplementary Table 4, Supplemental Digital Content 4**).**

**Figure 4.**
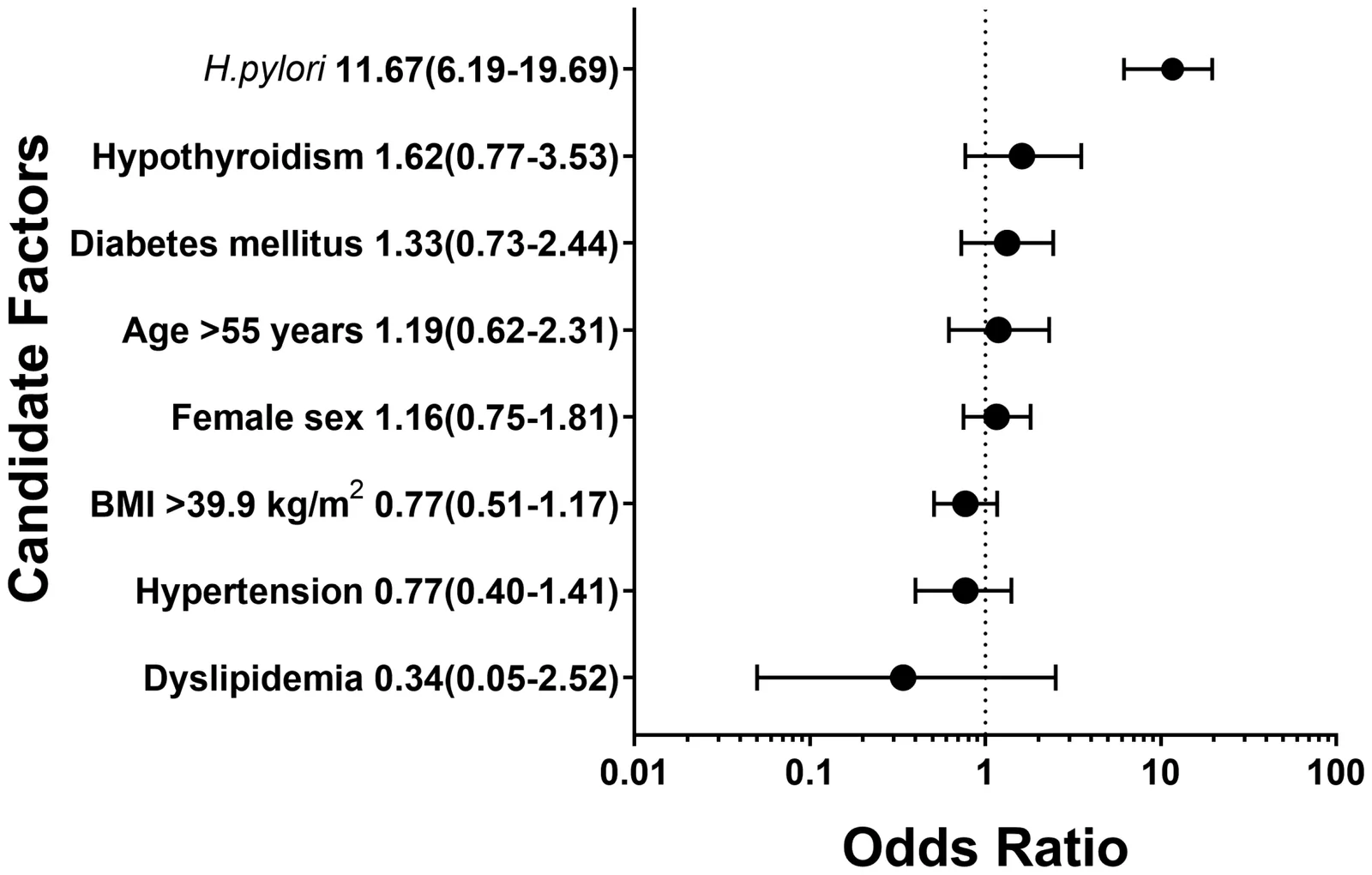
Unadjusted odds ratios (OR) with nominal 95% CIs for candidate factors associated with mild chronic active gastritis (103/954). *H. pylori* = present in the resected specimen. Fisher’s exact test; Bonferroni-adjusted threshold for 8 comparisons, α = 0.00625. BMI = body mass index, H. pylori = Helicobacter pylori, OR = odds ratio.

Figure 5 shows unadjusted OR for moderate chronic active gastritis (n = 20). *H. pylori* was strongly associated (OR 16.34, 95% CI 6.68–38.85; *P* < .001), which remained significant after Bonferroni adjustment (*α* = 0.00625) and again reflects co-occurrence in the same specimen. Age > 55 years showed a nominal association (OR 3.37, 95% CI 1.32–9.66; unadjusted *P* = .03) that did not remain significant after adjustment. No patient with dyslipidemia had this outcome (0/25 vs 20/929 without), so no OR is reported for it (Supplementary Table 5, Supplemental Digital Content 5).

**Figure 5.**
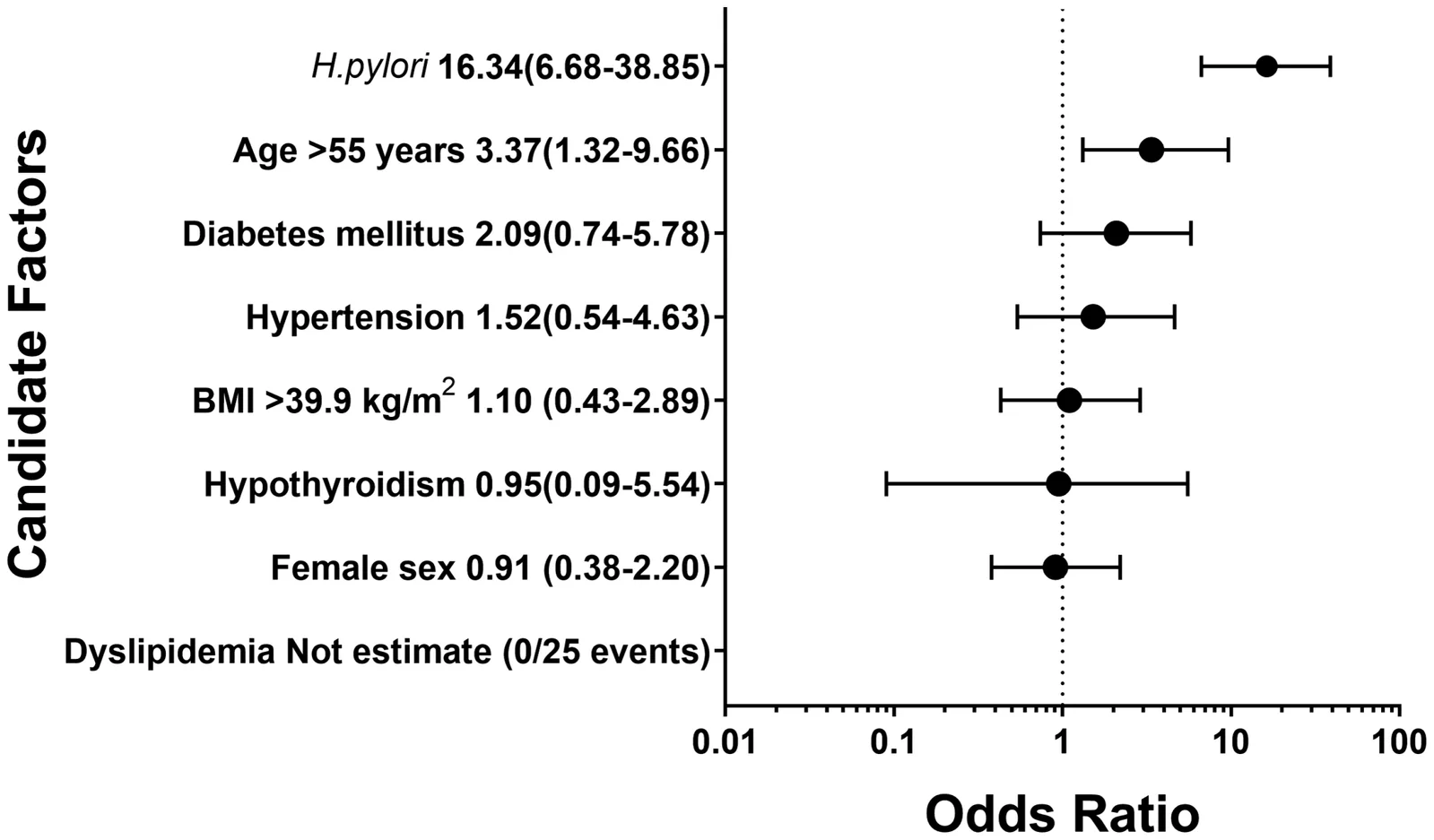
Unadjusted odds ratios (OR) with nominal 95% CIs for candidate factors associated with moderate chronic active gastritis (20/954). *H. pylori* = present in the resected specimen. Fisher’s exact test; Bonferroni-adjusted threshold for 8 comparisons, α = 0.00625. Dyslipidaemia: not estimable (no events). CI = confidence interval, H. pylori = Helicobacter pylori, OR = odds ratio.

Figure 6 shows unadjusted OR for follicular gastritis. No candidate factor was significantly associated with follicular gastritis, even before adjustment for multiple comparisons (all unadjusted *P* ≥ .25). BMI > 39.9 kg/m^2^ (OR 0.88, 95% CI 0.55–1.38; *P* = .63), *H. pylori* (OR 1.46, 95% CI 0.71–3.05; *P* = .29), and dyslipidemia (OR 1.45, 95% CI 0.45–4.76; *P* = .47) showed no association, and hypothyroidism had the lowest point estimate (OR 0.42, 95% CI 0.10–1.59; *P* = .31) (Supplementary Table 6, Supplemental Digital Content 6**).**

**Figure 6.**
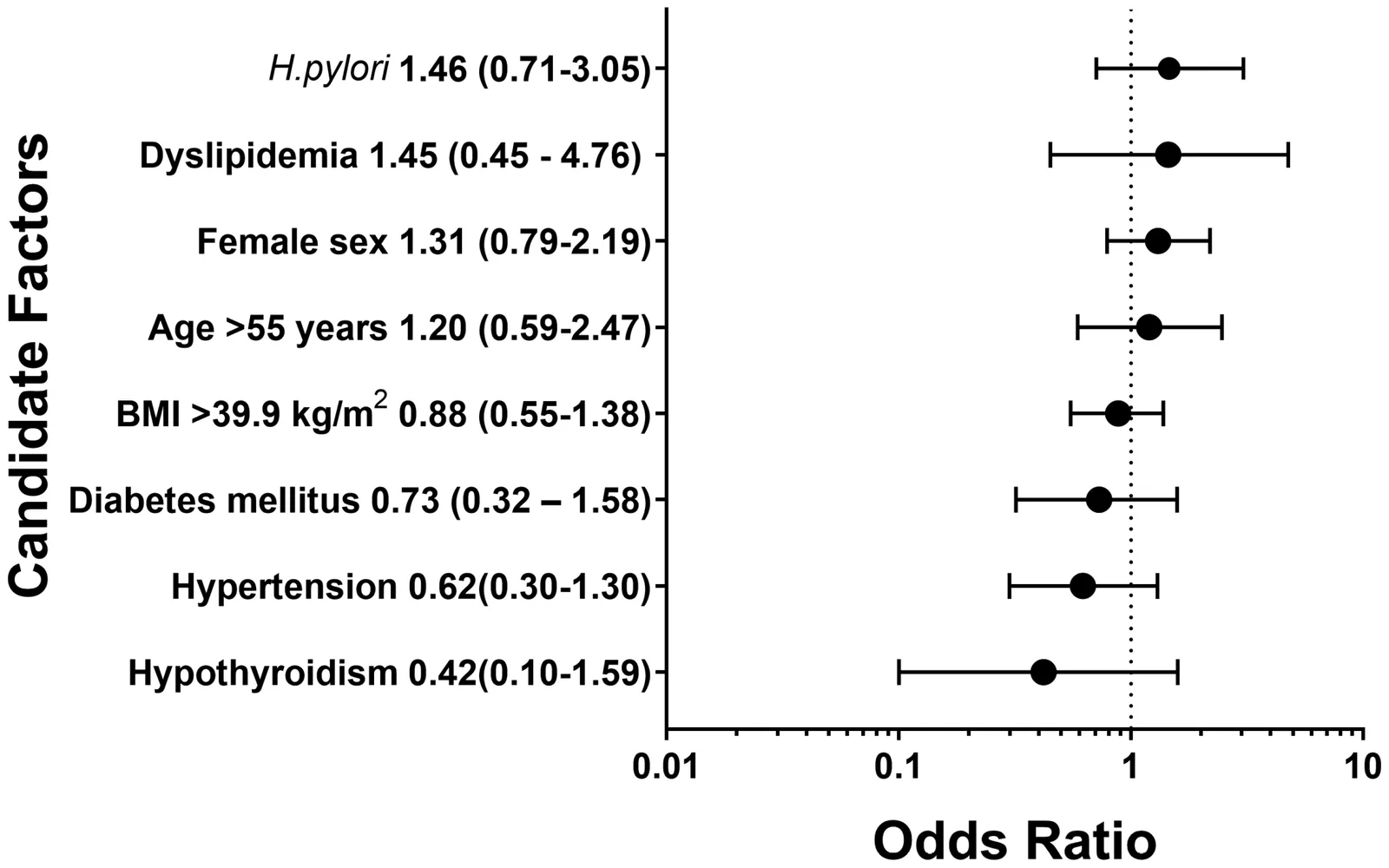
Unadjusted odds ratios (OR) with nominal 95% CIs for candidate factors associated with follicular gastritis (83/954). *H. pylori* = present in the resected specimen. Fisher’s exact test; Bonferroni-adjusted threshold for 8 comparisons, α = 0.00625. BMI = body mass index, H. pylori = Helicobacter pylori, OR = odds ratio.

## 4. Discussion

Among the patients who underwent LSG and had resected specimen histopathology, most were female. Our data align with results reported in numerous studies in the literature. Generally, there is a higher prevalence of females undergoing LSG than males. Demirbas, Erdim^[15]^ reported that a higher proportion of females (72%) underwent LSG than males (28%). Rashdan, Mansour^[16]^ also found that a greater proportion of females (77.5%) underwent sleeve gastrectomy. The mean age of the study group was 39.18. This was in agreement with Safaan and Bashah,^[17]^ *M*_*age*_ = 35.3, and Aykota and Yilmaz,^[18]^ *M*_*age*_ = 35.3. Keren, Matter^[19]^ documented similar results with subjects in age group 35 to 55 years, which is similar to our results. Most patients were classified as obese class III. Similar results were reported by Keren and Matter,^[19]^ Ahmed, Alanazi,^[20]^ and Tekeli and Eker.^[21]^

In our study, the most prevalent comorbidities were hypertension (14.3%), diabetes mellitus (10.9%), hypothyroidism (5.2%), bronchial asthma (2.7%), dyslipidemia (2.6%), and obstructive sleep apnea syndrome (2.2%). Keren, Matter^[19]^ reported the prevalence of diabetes mellitus to be 52.3%, hypertension at 41.46%, obstructive sleep apnea at 17.88%, joint pain at 65.85%, and GERD at 45.52%. The results were similar to our findings, but the discrepancy in percentages could be attributed to the difference in sample size, as the previous study included only 123 subjects. A previous study conducted by Padwal, Chang^[22]^ reported that the prevalence of dyslipidemia was 2%, hypertension was 13%, and type 2 diabetes was 21%.

LSG is currently regarded as the most popular form of bariatric surgery and is widely performed as one of the most common abdominal procedures. There is ongoing debate regarding the requirement for preoperative EGD before LSG, although the need remains uncertain.^[23]^ The European Association for Endoscopic Surgery and the Italian Society of Bariatric Surgery & Metabolic Diseases have issued recommendations for the routine use of preoperative EGD.^[24,25]^ In contrast, the American Society for Metabolic and Bariatric Surgery suggests selective preoperative EGD for symptomatic patients.^[26]^ Our study showed that only 37.4% of the patients included in our analysis underwent preoperative EGD, and 47.9% of these individuals exhibited normal examination results.

Among patients who underwent preoperative EGD, a normal EGD was associated with normal resected-specimen histopathology in most cases (NPV 99.4%), similar to observations by Canil, Iossa^[27]^ and Safaan, Bashah.^[17]^ However, this agreement was observed in a selected subgroup (37.4% of the cohort) and does not show that examination of the resected specimen can be omitted. EGD evaluates the mucosal surface, whereas histopathology examines the whole specimen microscopically. Gastritis was the most common abnormal finding on preoperative EGD; however, other abnormalities that may have implications for surgical decision-making are infrequent. Wiltberger, Bucher^[28]^ suggested that preoperative EGD is imperative, given the significance of the findings in relation to the surgical procedure and the potential to influence the treatment plan. For instance, certain studies have indicated that the presence of a sizable hiatal hernia or Barrett’s esophagus prior to surgery may necessitate a shift in the surgical strategy from LSG to Roux-en-Y gastric bypass.^[29]^ Therefore, preoperative EGD offers numerous benefits, including the timely identification of conditions that could impact surgical decision-making, such as severe esophagitis, Barrett’s esophagus, hiatal hernia, and tumors.

In our study, three patients were identified with GIST in the histopathological examination of their resected gastric specimens. All three were identified incidentally in resected specimens, and none of these patients had undergone preoperative EGD. Because GISTs are typically submucosal or intramural, EGD would not necessarily have detected them, so these cases neither indicate a missed opportunity nor support EGD as a substitute for histopathology. In contrast, Taha-Mehlitz, Mongelli^[30]^ contended that despite producing similar outcomes, preoperative EGD failed to detect 7.8% of pathologies that required further treatment and follow-up. This suggests that EGD and histopathology of the resected specimen may be complementary. Costs and clinical outcomes were not evaluated in this study.

In our study, gastritis was found to be the most common abnormality detected during preoperative EGD and in resected specimens post sleeve gastrectomy. These findings align with those of Taha-Mehlitz, Mongelli,^[30]^ who documented that gastritis was the most commonly occurring condition during preoperative EGD and in resected specimens after sleeve gastrectomy. For instance, Rashdan, Mansour^[16]^discovered that gastritis was the predominant histopathological observation in resected specimens after sleeve gastrectomy (88.3%). Numerous independent studies, including the study conducted by Salama, Saafan^[31]^ have arrived at a similar conclusion that gastritis (42.5%) is the chief histopathological alteration observed in resected gastric sleeves. The most frequently observed aberrant histopathologies in the resected gastric specimens were chronic inactive gastritis (22.6%), chronic active gastritis in both mild and moderate (12.9%), follicular gastritis (8.7%), and atrophic gastritis (0.2%). The same prevalence was reported by Safaan, Bashah^[17]^ for chronic inactive gastritis (33%), chronic active gastritis (6.8%), and follicular gastritis (2.7%).

AbdullGaffar, Raman^[32]^in the United Arab Emirates reported a prevalence of chronic gastritis at 45% and active gastritis at 8.4%. Almazeedi, Al-Sabah^[33]^ identified chronic gastritis as the most prevalent histopathological anomaly, accounting for 74.4% of all specimens. This was followed by follicular gastritis (9.6%), active follicular gastritis (4.9%), and atrophic gastritis (1.8%). These findings were consistent with those reported by Almazeedi, Al-Sabah.^[33]^ Nevertheless, the comparison of our results with those of Almazeedi, Al-Sabah^[33]^ and other studies was impeded by the use of distinct categories of aberrant histological alterations in various countries. For example, in our study, we identified follicular gastritis in 8.7% of our samples without differentiating between follicular and active follicular gastritis. However, Almazeedi, Al-Sabah^[33]^ identified 2 subtypes: follicular gastritis (9.6%) and active follicular gastritis (4.9%). The study also described chronic gastritis, with no distinction between active and inactive gastritis. Similarly, Raess, Baird-Howell^[34]^ reported gastritis (all subcategories, 12%) and chronic inflammation (12%). Thus, the use of several aberrant histopathological categories by researchers across different countries has made it challenging to accurately and directly compare the results among different studies. These results indicate the critical need for a more cohesive and uniform approach for categorizing aberrant histopathology.

In our unadjusted analysis, BMI > 39.9 kg/m^2^ was not significantly associated with gastritis (OR 1.27, 95% CI 0.81–1.93; *P* = .32), so larger studies are needed to clarify this. Yamamoto, Watabe^[35]^ and Aljerian^[36]^ proposed that obesity might be a risk factor for gastritis.

*H.pylori* is frequently identified in patients undergoing preoperative EGD, with a prevalence of 9.5% (34/357 EGD patients), and in histopathological analysis of resected specimens, with a prevalence of 8.0% (76/954). In unadjusted analyses, *H. pylori* was strongly associated with active chronic gastritis; the number of rare premalignant findings was too small to assess any association. This is consistent with previous studies conducted in Kuwait, the USA, and New Zealand, which reported prevalence rates of 7.3%, 5.2%, and 8.6%, respectively.^[33,34,37]^ Research conducted in Middle Eastern populations has demonstrated a significant prevalence of *H. pylori* infection, ranging from 20% to 97%.^[18]^ *H. pylori* is responsible for widespread chronic bacterial infection affecting approximately 50% of the global population, is associated with morbidity and mortality, and serves as a risk factor for gastritis, peptic ulcer, duodenitis, and other benign and malignant conditions.^[38]^ Despite the significance of *H. pylori* infection, its presence in preoperative EGD does not influence the postoperative outcomes of LSG Rossetti, Moccia.^[39]^ and Keren and Matter^[40]^ suggested that LSG may have the potential to eradicate *H. pylori* infection. The long-term implications of prolonged PPI use and of *H. pylori* eradication in this setting remain unresolved.

Our findings indicate that significant pathologies in resected specimens following sleeve gastrectomy, such as metaplasia, GIST, hyperplasia, gastric polyps, and GC grade 1, are infrequent. This observation aligns with the existing literature, which similarly reported a low incidence of serious findings. The clinical implications of these precancerous lesions have been explored previously. Gastric malignancy typically follows a multistep process, often progressing from normal mucosa to chronic gastritis, mucosal atrophy, intestinal metaplasia (characterized by the replacement of gastric epithelium with intestinal epithelium), dysplasia (intraepithelial neoplasia), and ultimately, cancer. This sequence of events, which may unfold over several years, is known as Correa’s cascade of multistep gastric carcinogenesis.^[41]^ Few studies have investigated the epidemiology of premalignant gastric lesions in asymptomatic individuals.

In 2011, a population-based study identified premalignant gastric lesions in 9.3% of asymptomatic individuals, with a higher prevalence in older patients (>60 years).^[42]^ In our cohort, the number of premalignant lesions was too small to assess their association with age. Previous studies have linked *H. pylori* infection with these conditions^[17,43–45]^; our data were too sparse to evaluate this. In a study by Rashdan and Mansour,^[16]^ metaplasia was observed in 2.2% of cases with no reported malignancies. Similarly, Salama and Saafan^[31]^ found metaplasia in 1.4% of patients and GIST in 0.7% of patients. A comparable study in Saudi Arabia reported tumor presence in only 1.2% of the patients.^[36]^ Matsuda and Li^[46]^ reported a substantial link between GIST and *H. pylori*, while Adiwinata and Andriani^[47]^ documented a connection between hyperplastic polyps and *H. pylori* infection. Chronic inflammation can lead to recurrent cell regeneration and proliferation. Atrophic gastritis, primarily caused by *H. pylori* infection, results in hypergastrinemia as a compensatory mechanism. Hypergastrinemia may create a favorable environment for cell growth and the formation of gastric polyps.^[48]^

Screening for precancerous lesions and monitoring individuals with gastric cancer may substantially reduce the incidence and mortality rates associated with this disease, which currently ranks as the third leading cause of cancer-related deaths.^[49]^ According to the guidelines established by the European Society of Gastrointestinal Endoscopy, patients exhibiting significant atrophy/metaplasia and dysplasia are considered optimal candidates for endoscopic gastric surveillance. The recommended surveillance intervals do not exceed three years for extensive atrophic gastritis and intestinal metaplasia, and do not exceed twelve months for low-grade dysplasia. Patients diagnosed with high-grade dysplasia should undergo a comprehensive evaluation and receive appropriate treatment.^[50]^

This study has the largest number of patients reported at the national level, and it is one of the largest internationally. However, it has several limitations. The retrospective design does not allow the follow-up of data necessary to evaluate the clinical or economic outcomes of histopathological findings, such as the progression of premalignant lesions or the impact of *H. pylori* eradication. Additionally, the study was conducted at a single center. Because the data were collected retrospectively from medical files, several details were not available in the files: the indications and selection criteria for EGD, the interval between EGD and surgery, whether gastric biopsies were taken and how *H. pylori* was diagnosed, eradication treatment and its confirmation, the sampling protocol for the resected specimens, the classification and criteria used for gastritis, the use of special stains or immunohistochemistry, the criteria used for GIST, carcinoid and premalignant lesions, and whether pathological assessment changed during the 13-year study period. Future prospective studies should record these details to allow replication. Another limitation is that only 37.4% of patients underwent preoperative EGD, which is not routinely performed and was not done under a standardized protocol; this indicates substantial selection bias, and the agreement reported applies only to this subgroup. Patients whose surgery was canceled after preoperative EGD are not represented because the cohort comprised patients who underwent LSG. EGD and resected-specimen histopathology assess different aspects of the stomach, so agreement does not imply equivalence, particularly for submucosal lesions such as GIST. Association analyses were unadjusted and exploratory, and rare outcomes could not be analyzed because of very few events. We recommend conducting a prospective study at multiple centers with predefined EGD indications, timing and data collection. This approach could produce more accurate data and facilitate a comprehensive correlation between preoperative EGD and resected specimen histopathology in patients undergoing LSG. In particular, the Ministry of Health in Saudi Arabia has accredited 20 facilities for obesity surgery and has incorporated follow-up data to assess the clinical implications of abnormal histological findings.

## 5. Conclusion

In conclusion, gastritis was the predominant histopathological finding in both preoperative EGD and resected specimens. In this retrospective single-center study, a normal preoperative EGD showed high agreement with normal resected-specimen histopathology in the selected subgroup who underwent EGD. Because of selection bias and the limitations above, our findings do not support omitting histopathological examination of the resected specimen. Prospective multicenter studies with predefined EGD indications, timing, and clinical and economic outcomes are needed. The incidence of neoplastic tumors in both the preoperative EGD and resected specimen histopathology was low. *H.pylori* was frequently detected by both preoperative EGD and resected-specimen histopathology and was strongly associated with gastritis in unadjusted analyses.

## Acknowledgements

This work was supported by Princess Nourah bint Abdulrahman University Researchers Supporting Project number (PNURSP2026R171), Princess Nourah bint Abdulrahman University, Riyadh, Saudi Arabia. This study was supported by the Researchers Supporting Project number (MHIRSP2025019), AlMaarefa University, Riyadh, Saudi Arabia.The authors deeply acknowledge the medical personnel and leadership of King Abdulaziz University, Jeddah, Saudi Arabia, who graciously supported this study.

## Author contributions

**Investigation:** Moaz Abulfaraj, Mohammed F. Alhazmi, Ashraf A. Maghrabi, Abdulrahman Faihan A. Alotaibi.

**Methodology:** Moaz Abulfaraj, Murad M. Aljiffry, Rana Y. Bokhary, Abdulrahman Faihan A. Alotaibi, Rania A. Fouad, Mohamed El-Sherbiny.

**Conceptualization:** Murad M. Aljiffry, Ashraf A. Maghrabi, Abdulrahman Faihan A. Alotaibi.

**Formal analysis:** Mohammed F. Alhazmi, Rana Y. Bokhary, Mohamed El-Sherbiny, Hasnaa Ali Ebrahim, Tagwa Salah Ahmed Mohammed Ali, Dalia Mahmoud Abdelmonem Elsherbini.

**Validation:** Mohammed F. Alhazmi.

**Data curation:** Ashraf A. Maghrabi, Rania A. Fouad.

**Funding acquisition:** Mohamed El-Sherbiny, Hasnaa Ali Ebrahim.

**Software:** Dalia Mahmoud Abdelmonem Elsherbini.

**Writing – original draft:** Moaz Abulfaraj, Rana Y. Bokhary, Abdulrahman Faihan A. Alotaibi, Mohamed El-Sherbiny, Dalia Mahmoud Abdelmonem Elsherbini.

**Writing – review & editing:** Murad M. Aljiffry, Rania A. Fouad, Hasnaa Ali Ebrahim, Tagwa Salah Ahmed Mohammed Ali, Dalia Mahmoud Abdelmonem Elsherbini.












