# Supplementary material for: Histopathological findings in laparoscopic sleeve gastrectomy specimens and their relation to preoperative endoscopy: A retrospective cross-sectional study of 954 patients
Source: Medicine (Baltimore). 2026 Oct 23;105(43):e51246. doi: 10.1097/MD.0000000000051246 (PMC13706432; doi:10.1097/MD.0000000000051246)
Supplement: Supplementary file 1 [file medi-105-e51246-s001.docx]

**Supplementary Table 1.** Unadjusted odds ratios and relative risks with 95% confidence intervals for candidate factors associated with gastritis on preoperative EGD (179/357)

| **Candidate factor** | **Odds Ratio**  **(95% CI)** | **Relative Risk**  **(95% CI)** | | ***p*-value**  Fisher's exact test |
| --- | --- | --- | --- | --- |
| Age > 55 years | 1.26 (0.65-2.53) | 1.12 (0.79-1.46) | | 0.61 |
| Female sex | 0.73 (0.46-1.15) | 0.86 (0.70-1.07) | | 0.20 |
| BMI >39.9 kg/m^2^ | 1.27 (0.81-1.93) | 1.13 (0.90-1.42) | | 0.32 |
| Hypertension | 1.19 (0.66-2.13) | 1.09(0.82-1.39) | | 0.65 |
| Diabetes mellitus | 1.05 (0.56-1.96) | 1.02 (0.72-1.35) | | 0.99 |
| Hypothyroidism | 1.00 (0.48-2.07) | 1.00(0.64-1.38) | | 0.99 |
| Dyslipidemia | 0.54 (0.18-1.64) | 0.70(0.35-1.43) | | 0.29 |
| *H. pylori*† | 87.7 (12.3–11,132)† | | 2.24 (1.98–2.52) | < 0.001*** |

† All 35 patients with *H. pylori* in the resected specimen had gastritis on preoperative EGD, so the odds ratio, its profile-likelihood 95% CI and the P value (penalised likelihood-ratio test) are from Firth penalised logistic regression; the relative risk is a conventional estimate *p****<0.001 is statistically significant after Bonferroni adjustment. P values are unadjusted (Fisher’s exact test); with eight comparisons per table the Bonferroni-adjusted threshold is α = 0.00625, so P < 0.05 without reaching it is a nominal association only. *H. pylori* = present in the resected specimen. EGD = esophagogastroduodenoscopy.
