# Supplementary material for: Histopathological findings in laparoscopic sleeve gastrectomy specimens and their relation to preoperative endoscopy: A retrospective cross-sectional study of 954 patients
Source: Medicine (Baltimore). 2026 Oct 23;105(43):e51246. doi: 10.1097/MD.0000000000051246 (PMC13706432; doi:10.1097/MD.0000000000051246)
Supplement: Supplementary file 2 [file medi-105-e51246-s002.docx]

**Supplementary Table 2.** Patients with more than one histopathological finding in the resected specimen

| **Combination of findings in one specimen** | **n** |
| --- | --- |
| **Gastritis with *H. pylori*** |  |
| Mild chronic active gastritis + *H. pylori* | 37 |
| Mild chronic inactive gastritis + *H. pylori* | 18 |
| Moderate chronic active gastritis + *H. pylori* | 10 |
| Follicular gastritis + *H. pylori* | 9 |
| **Gastritis with metaplasia** |  |
| Mild chronic inactive gastritis + metaplasia | 2 |
| Follicular gastritis + metaplasia | 1 |
| **Gastritis with hyperplasia** |  |
| Mild chronic inactive gastritis + hyperplasia | 1 |
| Chronic atrophic gastritis + hyperplasia | 1 |
| **Gastritis with focal erosion** |  |
| Follicular gastritis + focal erosion | 1 |
| **Gastritis with metaplasia and *H. pylori*** |  |
| Mild chronic inactive gastritis + metaplasia + *H. pylori* | 1 |
| Moderate chronic active gastritis + metaplasia + *H. pylori* | 1 |
| **Total patients with more than one finding** | **82 (8.6% of all patients; 18.9% of abnormal specimens)** |

Each patient appears once, under the combination of diagnoses recorded in the pathology report. Percentages are of all 954 patients and of the 434 patients with abnormal specimens. *H. pylori*: *Helicobacter pylori*.
