# Supplementary material for: Histopathological findings in laparoscopic sleeve gastrectomy specimens and their relation to preoperative endoscopy: A retrospective cross-sectional study of 954 patients
Source: Medicine (Baltimore). 2026 Oct 23;105(43):e51246. doi: 10.1097/MD.0000000000051246 (PMC13706432; doi:10.1097/MD.0000000000051246)
Supplement: Supplementary file 3 [file medi-105-e51246-s003.docx]

**Supplementary Table 3.** Unadjusted odds ratios and relative risks with 95% confidence intervals for candidate factors associated with mild chronic inactive gastritis (216/954)

| **Candidate factor** | **Odds Ratio**  **(95% CI)** | **Relative Risk**  **(95% CI)** | ***p*-value**  Fisher's exact test |
| --- | --- | --- | --- |
| Age > 55 years | 0.64 (0.36-1.11) | 0.70 (0.43-1.10) | 0.15 |
| Female sex | 1.00 (0.73-1.39) | 1.00 (0.78-1.29) | 0.98 |
| BMI >39.9 kg/m^2^ | 1.14 (0.84-1.56) | 1.11 (0.87-1.43) | 0.42 |
| Hypertension | 1.65(1.11-2.46) | 1.45(1.08-1.91) | 0.02 |
| Diabetes mellitus | 1.37 (0.88-2.17) | 1.27 (0.90-1.74) | 0.17 |
| Hypothyroidism | 1.08(0.56-2.09) | 1.06(0.63-1.69) | 0.86 |
| Dyslipidemia | 1.34 (0.52-3.27) | 1.25 (0.63-2.15) | 0.48 |
| *H.pylori* | 1.15(0.68-1.94) | 1.11(0.73-1.63) | 0.66 |

P values are unadjusted (Fisher’s exact test); with eight comparisons per table the Bonferroni-adjusted threshold is α = 0.00625, so P < 0.05 without reaching it (hypertension, P = 0.02) is a nominal association only. *H. pylori* = present in the resected specimen.
