# Supplementary material for: Histopathological findings in laparoscopic sleeve gastrectomy specimens and their relation to preoperative endoscopy: A retrospective cross-sectional study of 954 patients
Source: Medicine (Baltimore). 2026 Oct 23;105(43):e51246. doi: 10.1097/MD.0000000000051246 (PMC13706432; doi:10.1097/MD.0000000000051246)
Supplement: Supplementary file 4 [file medi-105-e51246-s004.docx]

**Supplementary Table 4.** Unadjusted odds ratios and relative risks with 95% confidence intervals for candidate factors associated with mild chronic active gastritis (103/954)

| **Candidate factor** | **Odds Ratio**  **(95% CI)** | **Relative Risk**  **(95% CI)** | ***p*-value**  Fisher's exact test |
| --- | --- | --- | --- |
| Age > 55 years | 1.19 (0.62-2.31) | 1.16 (0.64-2.03) | 0.59 |
| Female sex | 1.16 (0.75-1.81) | 1.14 (0.77-1.70) | 0.58 |
| BMI >39.9 kg/m^2^ | 0.77 (0.51-1.17) | 0.79 (0.55-1.14) | 0.24 |
| Hypertension | 0.77(0.40-1.41) | 0.80(0.45-1.38) | 0.55 |
| Diabetes mellitus | 1.33(0.73-2.44) | 1.29(0.76-2.13) | 0.40 |
| Hypothyroidism | 1.62(0.77-3.53) | 1.52(0.77-2.80) | 0.24 |
| Dyslipidemia | 0.34 (0.05-2.52) | 0.36 (0.05-2.51) | 0.51 |
| *H.pylori* | 11.67(6.19-19.69) | 6.48(4.61-8.88) | < 0.001*** |

*p****<0.001 is significant after Bonferroni adjustment. P values are unadjusted (Fisher’s exact test); with eight comparisons per table the Bonferroni-adjusted threshold is α = 0.00625, so P < 0.05 without reaching it is a nominal association only. *H. pylori* = present in the resected specimen.
