# Supplementary material for: Histopathological findings in laparoscopic sleeve gastrectomy specimens and their relation to preoperative endoscopy: A retrospective cross-sectional study of 954 patients
Source: Medicine (Baltimore). 2026 Oct 23;105(43):e51246. doi: 10.1097/MD.0000000000051246 (PMC13706432; doi:10.1097/MD.0000000000051246)
Supplement: Supplementary file 5 [file medi-105-e51246-s005.docx]

**Supplementary Table 5.** Unadjusted odds ratios and relative risks with 95% confidence intervals for candidate factors associated with moderate chronic active gastritis (20/954)

| **Candidate factor** | **Odds Ratio**  **(95% CI)** | **Relative Risk**  **(95% CI)** | ***p*-value**  Fisher's exact test |
| --- | --- | --- | --- |
| Age > 55 years | 3.37 (1.32-9.66) | 3.24 (1.24-8.27) | 0.03 |
| Female sex | 0.91 (0.38-2.20) | 0.91 (0.38-2.20) | 0.81 |
| BMI >39.9 kg/m^2^ | 1.10 (0.43-2.89) | 1.10 (0.46-2.65) | 0.98 |
| Hypertension | 1.52(0.54-4.63) | 1.50(0.53-4.18) | 0.51 |
| Diabetes mellitus | 2.09(0.74-5.78) | 2.04(0.72-5.64) | 0.26 |
| Hypothyroidism | 0.95(0.09-5.54) | 0.95(0.16-5.25) | 0.98 |
| Dyslipidemia† | ------------------ | ------------------- | 1.00 |
| *H.pylori* | 16.34(6.68-38.85) | 14.12(6.13-32.02) | < 0.001*** |

† No patient with dyslipidaemia had this outcome (0/25 versus 20/929); with fewer than one event expected in this group, the odds ratio and relative risk are not estimable. *p****<0.001 is Significant after Bonferroni adjustment. P values are unadjusted (Fisher’s exact test); with eight comparisons per table the Bonferroni-adjusted threshold is α = 0.00625, so P < 0.05 without reaching it (age >55 years, P = 0.03) is a nominal association only. *H. pylori* = present in the resected specimen.
