# Supplementary material for: Histopathological findings in laparoscopic sleeve gastrectomy specimens and their relation to preoperative endoscopy: A retrospective cross-sectional study of 954 patients
Source: Medicine (Baltimore). 2026 Oct 23;105(43):e51246. doi: 10.1097/MD.0000000000051246 (PMC13706432; doi:10.1097/MD.0000000000051246)
Supplement: Supplementary file 6 [file medi-105-e51246-s006.docx]

**Supplementary Table 6.** Unadjusted odds ratios and relative risks with 95% confidence intervals for candidate factors associated with follicular gastritis (83/954)

| **Candidate factor** | **Odds Ratio**  **(95% CI)** | **Relative Risk**  **(95% CI)** | ***p*-value**  Fisher's exact test |
| --- | --- | --- | --- |
| Age > 55 years | 1.20 (0.59-2.47) | 1.18 (0.61-2.20) | 0.56 |
| Female sex | 1.31 (0.79-2.19) | 1.28 (0.81 – 2.02) | 0.33 |
| BMI >39.9 kg/m^2^ | 0.88 (0.55-1.38) | 0.89 (0.59-1.36) | 0.63 |
| Hypertension | 0.62(0.30-1.30) | 0.64(0.32-1.26) | 0.25 |
| Diabetes mellitus | 0.73 (0.32 – 1.58) | 0.75 (0.36 – 1.53) | 0.58 |
| Hypothyroidism | 0.42(0.10 – 1.59) | 0.45(0.12-1.52) | 0.31 |
| Dyslipidemia | 1.45 (0.45 – 4.76) | 1.40 (0.48 – 3.58) | 0.47 |
| *H.pylorii* | 1.46(0.71-3.05) | 1.41(0.73-2.60) | 0.29 |

P values are unadjusted (Fisher’s exact test); with eight comparisons per table the Bonferroni-adjusted threshold is α = 0.00625, so P < 0.05 without reaching it is a nominal association only. H. pylori = present in the resected specimen.
